# ﻿*Chaetophilosciasicula* Verhoeff, 1908 (Isopoda, Oniscidea), an invasive isopod currently spreading in North America

**DOI:** 10.3897/zookeys.1225.120937

**Published:** 2025-02-05

**Authors:** Katalin Szlavecz, Nathan T. Jones, Franck Noël, Pepijn Boeraeve, Pallieter De Smedt

**Affiliations:** 1 Department of Earth and Planetary Sciences, Johns Hopkins University, Baltimore, MD, USA Johns Hopkins University Baltimore United States of America; 2 American Isopod and Myriapod Group, Virginia, USA American Isopod and Myriapod Group Virginia United States of America; 3 Saint-Martin-de-Connée, France Unaffiliated Saint-Martin-de-Connée France; 4 Spinicornis, Bonheiden, Belgium Spinicornis Bonheiden Belgium; 5 Forest & Nature Lab, Ghent University, Gontrode, Belgium Ghent University Gontrode Belgium

**Keywords:** Introduction, non-native species, pet trade, urban habitats, woodlice

## Abstract

*Chaetophilosciasicula* Verhoeff, 1908 (Philosciidae) is a small terrestrial isopod of Mediterranean origin which was first reported in North America in 2000 in an urban forest in Baltimore, Maryland, and it was thought to be a recent introduction, with a restricted range. Here we report the current state of knowledge of *C.sicula* distribution in North America. Since the original observation, the species has been reported by citizen scientists from eight additional states. Standardized field surveys in Maryland and Washington D.C. revealed a strong habitat preference towards anthropogenic and coastal areas. The affinity of *C.sicula* to urban environments, including residential areas and urban parks, is reinforced by citizen-science data and is most likely key to its fast spread throughout North America. Keeping isopods as pets and trading them among hobbyists may also play a role especially in establishing core populations in urban centers. The species is likely to expand in the USA in the coming decade. This study highlights that thorough, systematic surveys, using a variety of collecting techniques, are essential to address existing knowledge gaps on terrestrial isopod distribution and spread in North America and elsewhere.

## ﻿Introduction

Humans have been transporting species beyond their historical ranges for centuries, and these intentional and unintentional introductions often negatively affected native biodiversity and ecosystem functions. In North America, many soil invertebrates that were introduced and established a long time ago (Lindroth 1957) have been invading wildland habitats where they potentially alter soil-microbial community structure, understory vegetation, and decomposition and nutrient cycling pathways. Extensive research has shown this to be the case for earthworm-invaded areas (e.g., Szlavecz et al. 2011; Ferlian et al. 2018; Chang et al. 2021). However, only a handful of studies, such as by Frouz et al. (2008) on the isopod *Armadillidiumvulgare* and MacLeod et al. (2024) on the larvae of the invasive Japanese beetle *Popilliajaponica*, have demonstrated that other invasive soil organisms can also significantly affect ecosystem properties. Proper assessment of potential risks by species invasions requires 1) accurate species identification, 2) sound knowledge on native and introduced distribution ranges, and 3) systematic monitoring to estimate spread (Pagad et al. 2018). In North America, huge data gaps exist in all three requirements for most non-native saprophagous soil macro- and mesofauna.

The terrestrial isopod fauna of North America is dominated by non-native species, especially at mid- to high latitudes. Native species are restricted to coastal and wetland areas, caves, and arid regions at lower latitudes (Jass and Klausmeier 2000; Shultz 2018). The most common non-native species had arrived with the early European settlers (Lindroth 1957) and spread throughout the continent (e.g., Garthwaite et al. 1995). Many species have reached extremely high abundances in a variety of habitats, including mesic and hydric forests (Frouz et al. 2004; Hornung et al. 2015), grasslands (Paris and Pitelka 1962), lake shorelines (Hatchett 1947), and residential areas (Philpott et al. 2014; Szlavecz et al. 2018).

**Figure 1.**
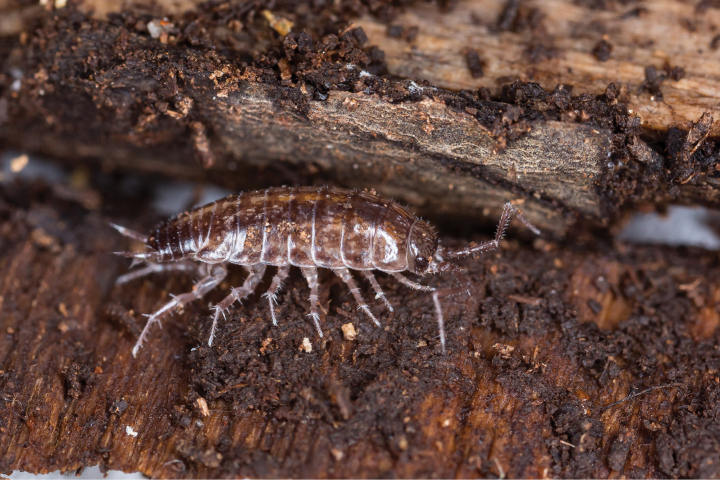
*Chaetophilosciasicula* Verhoeff, 1908 collected in Baltimore, Maryland, in a pile of wood chips in May 2023. The specimen in the picture is about 6 mm long. © Spinicornis – Gert Arijs.

Two decades ago, *Chaetophilosciasicula* Verhoeff, 1908 (Philosciidae, Fig. 1) was reported in Baltimore, Maryland, for the first time from North America (Hornung and Szlavecz 2003). It was found in an urban forest patch near a housing complex and later on the Homewood campus of Johns Hopkins University. *Chaetophilosciasicula* was thought to be a recent introduction with a restricted range. Since 2017, an increasing number of observations on the citizen-science platform iNaturalist (2024) has indicated that the species might be more common and widespread than previously thought. Using data from a recent, extensive field survey in the state of Maryland, where it was first recorded, and verified records of iNaturalist (2024), we map the current distribution of the species. The goal is to assess habitat preferences and invasion potential of this recent introduction into North America.

## ﻿Methods

To assess the current distribution of *C.sicula* we (1) collected data during a large field survey campaign to assess soil arthropod distribution in the state of Maryland and Washington D.C. and (2) extracted community science data from iNaturalist (2024).

**Figure 2.**
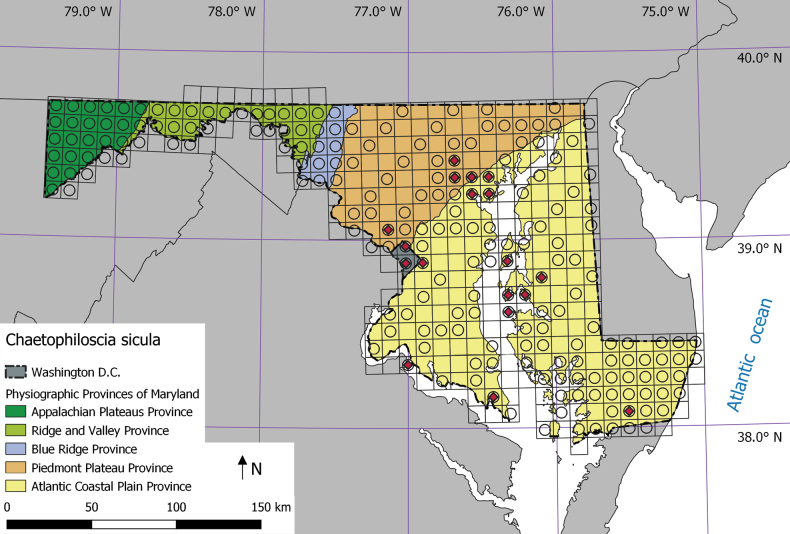
Distribution map of *Chaetophilosciasicula* in Maryland and Washington D.C, USA. Squares are 10 km × 10 km. Circles represent the visited squares and red diamonds indicate the presence of *C.sicula*. Physiographic provinces follow Reger and Cleaves (2008).

The field campaign was conducted across the state of Maryland and Washington D.C between July and November 2022, with additional surveys in May 2023. We selected this area due to its topographic, physiographic, and ecological diversity, with flat coastal plains in the south-east towards more mountainous forested areas in the west. Surveying a variety of ecosystems allowed us to gain insight into the habitat preferences of the species. Following the methodology of Boeraeve et al. (2022), the study area was divided into 10 km × 10 km UTM-squares, 367 squares in total (Fig. 2). To get a good representation of all ecological regions, every square in the western and eastern part of the state was sampled, while in the middle region every third square was sampled (Fig. 2). Within each square, at least two out of the following four habitat types were visited: forest, open habitat, anthropogenic habitat, and coastal habitat. If present, cave habitats were also visited. At each location isopods were searched for 1 h by sifting leaf litter, compost, or mulch or turning over dead wood, stones and other cover objects. We also recorded environmental variables such as canopy cover and the vicinity of water bodies. In total, 374 localities were searched for terrestrial isopods of which 148 forests, 34 open landscapes, 130 anthropogenic habitats, 53 coastal habitats, and nine caves. Individuals of *C.sicula* were preserved in 70% ethanol and taken to the laboratory for verification and sex determination.

iNaturalist observations (iNaturalist 2024) of *C.sicula* were verified by the authors based on “Research Grade” and “Needs ID” quality grade observations through habitus photos of live individuals between November 2017 and February 2024. Location data was exported using the iNaturalist website’s built-in export tool on February 11, 2024.

Maps were produced in QGis version 3.4.6. Locality information is provided in the Suppl. material 1.

## ﻿Results

During the structured field surveys, we collected *Chaetophilosciasicula* at 32 locations (8.5% of all locations). In all cases it was collected with other cosmopolitan species, including *Armadillidiumnasatum* (25 out of 32 locations), *Philosciamuscorum* (24 locations), *Hyloniscusriparius* (21 locations), *Armadilliddiumvulgare* (18 locations), and *Haplophthalmusdanicus* (14 locations). Where the species was present, we collected between one and 26 individuals (median 5.5) for a total of 240 individuals. Of these, 214 individuals were adults, with 71 individuals (33.2%) being males. Geographically, the species was only found in the Piedmont Plateau Province and the Atlantic Coastal Plain Province (Fig. 2). The species shows a pronounced habitat preference (Table 1), with 56% percent of the observations made in anthropogenic habitat, 34% in coastal habitat (seashores), and 9% (only three observations) in forests. We did not find *C.sicula* in either open landscape habitat or caves. *Chaetophilosciasicula* was found in 13.6% of all sampled anthropogenic locations and in 20.8% of all coastal locations. The majority of anthropogenic records were from gardens and public parks with no or an open canopy. The coastal locations were almost always the upper parts of sandy beaches under driftwood and other debris. A few locations were under debris between stones of jetties. Coastal locations were open with little or no shade from trees. Most coastal locations were close to anthropogenic environments. Abundances in anthropogenic and coastal habitat were often high with tens of individuals collected while sieving one litter sample. All coastal locations where *C.sicula* was recorded were in the Chesapeake Bay while no records were done at the coast of the Atlantic Ocean. The three observations in forested habitats were in urban forests in Baltimore or Washington D.C. These deciduous forests were relatively open with a maximum of 75% canopy cover. Only a few individuals were found in each forest and all of them were collected close to a stream.

**Table 1.** Distribution of *Chaetophilosciasicula* records by habitat types. Data from the structured survey in Maryland and Washington D.C. The species was not recorded in any of the nine caves included in the survey.

	Forest	Anthropogenic	Coast	Open landscape
Number of locations with *C.sicula*	3	18	11	0
Number of locations sampled	139	132	53	34
Percentage of locations with *C.sicula*	2.2	13.6	20.8	0

**Figure 3.**
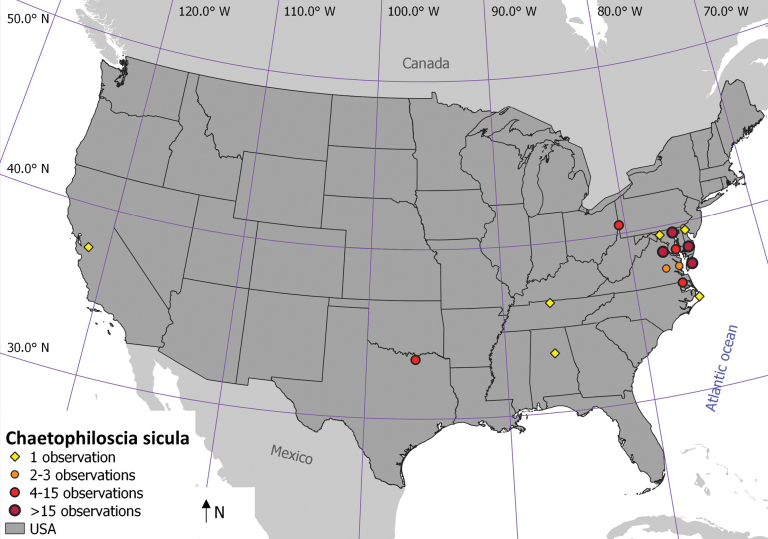
Distribution of *Chaetophilosciasicula* in the USA as of February 2024. Symbols indicate the number of observations (both iNaturalist and field survey observations) in 1 degree by 1 degree grid cells.

We identified 140 records of *C.sicula* from iNaturalist from nine states and Washington D.C. (Fig. 3). The majority of the records were from Virginia (80) and Maryland (37). We document the species for the first time in Virginia (first record in 2017), Texas (2018), Tennessee (2021), New Jersey (2021), California (2022), North Carolina (2022), Alabama (2023), and Pennsylvania (2023), and present multiple records from Washington D.C. (earliest from 2017). The sighting in California is interesting and indicates that the distribution of the species has also expanded to the US west coast.

In addition to the identification based on photographs, several Alabama, Virginia, and Texas specimens were verified based on the dissection of male sexual characteristics. All but one records are from anthropogenic habitat. For two observations, no habitat data could be retrieved, as the observer had obscured it. As for the structured field surveys, most anthropogenic records are from private gardens and public parks. At least 58 of the 140 locations are close to a stream or water body. The record from North Carolina represents the only coastal record along the Atlantic coast so far.

## ﻿Discussion

*Chaetophilosciasicula* is clearly spreading across North America. It appears to be most common around Baltimore where it was first recorded (Hornung and Szlavecz 2003). It has become widespread in the states of Maryland and Virginia, but there are recent records from at least seven other US states. This is probably an underestimation since the over 113,000 terrestrial isopod records on the iNaturalist platform, many still not validated, may yield new records in additional states (iNaturalist 2024). Although most of the records in the seven states are isolated, the distribution and habitat preference in Maryland indicate that the species can establish and spread from isolated localities. With a first record from the west coast, it can be expected that the species will colonize the entire United States. During the last three years, the species was recorded in two additional states every year.

We are aware of issues regarding misidentification and the pitfalls using iNaturalist data for scientific research (Barbato et al. 2021; Munzi et al. 2023). The problem is tangible especially for taxonomically difficult groups as is the case for many soil invertebrate taxa. For instance, the California observation of *C.sicula* was originally misidentified as the genus *Ligidium*. Expert validation is crucial here, which is what we provided by only examining high quality photographs and by additional dissections of the male specimen.

Most locations are in or nearby populated areas. While this may indicate human bias, i.e., people preferentially search in green spaces near their residences, we can still safely assume that human-aided transport is the most important way of spreading. Our records also suggest that *C.sicula* can establish populations in more natural systems such as coastal areas and (urban) forests. The species tolerates salt spray well; humid conditions along coastlines and forested habitats can facilitate natural spread of the species. *Chaetophilosciasicula* is small and, like other philosciid isopods, probably drought sensitive (Dias et al. 2013); thus they depend on moist conditions to spread. Many observations from riparian areas appear to confirm this and also suggest that stream banks could act as natural corridors for active dispersal.

Interestingly, the species is also rapidly spreading in Europe outside its native range. *Chaetophilosciasicula* originates from the northern Mediterranean region and occurs from northeastern Spain to Greece (Schmalfuss 2003). Recent records suggest that it is expanding not only within its native range, but beyond it. In Spain it appears to spread southwards with new observations in the central regions up to Madrid. In France it is rapidly expanding westward, from the Mediterranean coast towards the Atlantic coast, and north-east, with recent records from Paris, Dijon and Châlons-en-Champagne (Noël et al. 2014; Noël in press). New records indicating eastward expansion have also been reported. While *C.sicula* has not been found in Slovenia (Vittori et al. 2023), Giurginca and Vănoaica (2000) and Giurginca (2022) reported it from Romania. Turbanov and Gongalsky (2014) reported the species in Sevastopol, the Crimean Peninsula. Two recent observations were also reported in Sevastopol and Simferopol (iNaturalist 2024). In Great Britain, *C.sicula* was recently discovered but appears to be restricted to heated greenhouses (Gregory 2014). While naturally occurring in alluvial plains, meadows, and other open habitats (Cruz 1991; Taiti and Argano 2011; Noël et al. 2014), records from newly colonized locations are often from garden centers and nurseries. These new and isolated anthropogenic records in Europe underpin the importance of human-aided dispersal for *C.sicula*, highlighting similar mechanisms on both continents. In Europe a similar expansion is occurring by *Armadillidiumarcangelii*, another Mediterranean species that is rapidly spreading to new areas via potted plants (Noël et al. 2022; De Smedt and Van Dijck 2023). These examples illustrate how fast soil invertebrates can spread in just a few years when facilitated by human activities.

In addition to unintentional transport in North America and elsewhere, increasing national and international pet trade might further facilitate the rapid spread of *C.sicula*. In recent decades, keeping isopods as pets has gained significant popularity with tens of thousands of breeders in the US alone looking for colorful and rare species to breed (De Smedt et al. submitted). A few years ago, a supposedly native American isopod was collected from Alabama and sold nationally under the name *Rhyscotustexensis*. The individuals were later correctly identified as *C.sicula*. When this came to light, vendors culled their populations to prevent its spread; as a result, the species virtually disappeared from the trade (personal observations). However, intentional and accidental release of *C.sicula* colonies might have followed contributing to the increased sightings in other states.

Despite strict restrictions on live specimen import and tens of thousands of interceptions at the US borders annually (Turner et al. 2021), species introductions are still happening as the current cases for Emerald ash borer (*Agrilusplanipennis* Fairmaire, 1888) and the agricultural pest *Thripsparvispinus* (Thysanoptera, Thripidae) indicate (Poland and McCullough 2006; Ataide et al. 2024). Small organisms are unnoticed and/or illegally smuggled into the country. Soil organisms as potential invasive species might be overlooked as well, because soil import is heavily regulated, which, among other things, requires that only designated laboratories can receive foreign soil and strict protocols are in place for processing and discarding samples.

In conclusion, *C.sicula* successfully established in North America, and it is currently spreading. The species might have been around for longer than originally thought but was undetected due to its smaller size and lack of effort to systematically assess soil fauna in various ecosystems. As for consequences of new species invasions, in the absence of information on specific traits, one can only speculate. *Chaetophilosciasicula* is common and abundant in residential areas, potentially becoming a nuisance species. Another philosciid species of similar size, *Pulmoniscusturbanaensis*, is currently invading Colombia, South America, and causing problems in buildings and in small family farms (López-Orozco et al. 2017; De Smedt et al. submitted). Hornung and Szlavecz (2003) provided some characteristics of the Baltimore population. *Chaetophilosciasicula* exhibited highly clumped dispersion with a mean density of 22.7 individuals m^−2^. Median body lengths of females and males are 5.9 and 4.3 mm, respectively. Reproductive peak is in June and July with 7–18 eggs in the marsupium depending on female size. Comparison of characteristics well as genetic similarity of these disjunct populations both in North America and in Europe may shed light on the mechanisms of introduction and spread.

Based upon our data, we propose to add *C.sicula* to the list of synanthropic species (Jass and Klausmeier 2000) that dominate the isopod fauna of North America. This study highlights the need for more systematic faunal surveys in the US which will provide a clearer picture on the shifting ranges of native and introduced species.

## ﻿Additional information

### Author ORCIDs

Katalin Szlavecz https://orcid.org/0000-0003-2504-0298

Nathan T. Jones https://orcid.org/0000-0002-2365-6980

Pepijn Boeraeve https://orcid.org/0000-0002-3752-6407

Pallieter De Smedt https://orcid.org/0000-0002-3073-6751

### Data availability

All of the data that support the findings of this study are available in the main text or Supplementary Information.

## Supplementary materials

Supplementary material 1 Localities of *Chaetophilosciasicula* in North America Data type xlsx https://binary.pensoft.net/file/1242890This dataset is made available under the Open Database License (http://opendatacommons.org/licenses/odbl/1.0/). The Open Database License (ODbL) is a license agreement intended to allow users to freely share, modify, and use this Dataset while maintaining this same freedom for others, provided that the original source and author(s) are credited.Katalin Szlavecz, Nathan T. Jones, Franck Noël, Pepijn Boeraeve, Pallieter De Smedt
